# In Situ Heating STEM of the Thickness‐Dependent Evolution of Ferroelastic Domains

**DOI:** 10.1002/smll.74540

**Published:** 2026-07-14

**Authors:** Luka Geddis Zellmann, Sumner B. Harris, John J. R. Scott, Yi‐Chieh Yang, Joerg R. Jinschek, Rama K. Vasudevan, Miryam Arredondo

**Affiliations:** ^1^ School of Mathematics and Physics Queen's University Belfast Belfast UK; ^2^ Center for Nanophase Materials Sciences (CNMS) Oak Ridge National Laboratory (ORNL) Oak Ridge Tennessee USA; ^3^ National Center For Nano Fabrication and Characterization (DTU Nanolab) Technical University of Denmark Kongens Lyngby Denmark

**Keywords:** domain wall curvature, domains, ferroelastics, in situ heating, machine learning, scanning transmission electron microscopy

## Abstract

Ferroelastic domain walls (DWs) underpin key functionalities in complex oxides, where their behavior is governed by elastic interactions and boundary conditions. In ferroic thin films, these interactions are strongly thickness dependent, characterized by a monopolar‐dipolar crossover in DW behavior. However, how temperature influences DW dynamics within these size‐controlled interaction regimes remains unexplored. Here, LaAlO_3_ thin films spanning the dipolar (< 200 nm) and crossover (200–300 nm) regimes are investigated using in situ heating scanning transmission electron microscopy (STEM) and a machine learning‐driven image analysis approach. By tracking DW curvature and area fraction during cooling from above *T_C_* (∼550°C) to room temperature (RT), we reveal a distinct interplay between temperature and thickness. In the dipolar regime, DWs exhibit large curvature and significant reconfiguration near *T_C_*, which reduces upon cooling, consistent with the well‐known temperature freezing regime. In contrast, within the crossover regime, DWs remain nearly immobile with minimal reconfiguration through cooling and curvature values an order of magnitude lower at RT. These results map the evolution of DWs across the thermally driven super‐elastic to freezing regimes, revealing the interplay between elastic interaction regimes and thermal activation, providing insights for domain engineering in free‐standing oxide thin films.

## Graphical abstract

In‐situ heating scanning transmission electron microscopy combined with machine‐learning segmentation is used to study the thickness and temperature dependent behavior of ferroelastic domain walls in free‐standing LaAlO_3_ samples. The results quantify domain wall curvature and density throughout cooling, revealing a pronounced thickness‐dependent transition from highly mobile, curved domain walls to frozen, low‐curvature configurations.

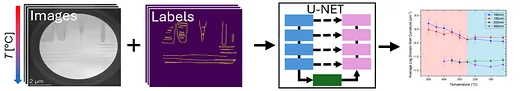

## 1 Introduction

Ferroelastic materials, like other ferroics, exhibit regions of uniform order parameter orientation known as domains that are separated by domain walls (DWs), and which can be switched by the application of external fields. Ferroelastic domains originate from spontaneous strain, with DWs accommodating lattice distortions between differently oriented domains. Beyond serving as passive strain boundaries, DWs are recognized as active functional entities with emergent physical properties [1] such as polarity [2, 3] and conductivity [4, 5]. This concept is referred to as “domain boundary engineering” [6], where the structure properties and dynamics of DWs can be exploited as nanoscale templates for devices [7, 8]. This approach offers routes to emergent properties with potential for novel applications in neuromorphic computing [9, 10], multiferroic devices [11, 12], and adaptive electronics [13].

In ferroelastics such as the model system LaAlO_3_ (LAO), DW dynamics are governed by strain‐mediated interactions, defect pinning, and long‐range elastic forces [14]. Salje and Lu's model established that kinks (atomic steps contained within DWs) generate long‐range elastic strain fields that mediate interactions between DWs. In this framework, the interaction energy depends on the DW separation (*d*), exhibiting dipolar scaling (*∼d^–^^2^*) when elastic relaxations are allowed, and monopolar scaling (*∼d^–^^1^)* when such relaxations are suppressed. This leads to a size‐dependent monopolar–dipolar crossover regime around a critical system size [15, 16]. In these models, the system is described as a finite strip with thickness (*t*), where DWs extend through the thickness, and the wall‐wall separation (*d*) is defined in‐plane, normal to the DWs. Importantly, the crossover between dipolar and monopolar interactions is governed by the ability of the system to elastically relax. Kink‐induced strain fields extend throughout the sample, and when boundary conditions allow macroscopic relaxation, these strain fields result in dipolar interactions between DWs, leading to DW bending and warping. When such relaxation is suppressed (e.g., due to increased system size or clamping), monopolar behavior emerges.

This framework has been experimentally validated by scanning transmission electron microscopy (STEM) studies at room temperature (RT), where a thickness‐driven crossover regime was identified, with thinner samples exhibiting increased DW curvature [17]. While these studies revealed the static, thickness‐dependent domain behavior, the thermally driven DW behavior within the crossover regime is still largely unexplored. In bulk ferroelastics, such as LAO, temperature plays a critical role, controlling wall mobility and defect pinning through two distinct temperature regimes: a super‐elastic regime at elevated temperatures, where DWs are highly mobile and responsive, and a freezing regime where DWs become pinned by defects and mobility is suppressed [18]. Additionally, temperature‐dependent studies in bulk LAO demonstrated that geometrical features, such as aspect ratio, strongly influence domain and DW evolution [19].

However, how thermal activation and size effects couple, and whether temperature can be leveraged to tune DW morphology and evolution remains unknown. This coupling would be particularly significant at the nanoscale, where effects such as DW curvature and junction formation are amplified. Here, we investigate how the DW behavior within these thickness‐dependent elastic interaction regimes evolves with temperature.

We examine how temperature influences DW response across thickness in free‐standing LAO films spanning the monopolar‐dipolar regimes, with particular focus on the crossover regime. LAO is selected as a model pure ferroelastic system due to its well‐characterized domain structure and previous work [17, 19]. Using in situ heating STEM combined with machine learning–based image segmentation, we quantify changes in DW area fraction and curvature during cooling from above the phase transition (*T_C_*). This study demonstrates a strong coupling between thickness and temperature, revealing that these parameters can be used to control DW morphology and evolution, establishing a foundation for domain boundary engineering in free‐standing oxide membranes and new routes toward adaptive domain‐engineered devices.

## 2 Results and Discussion

Free‐standing LaAlO_3_ (LAO) samples with thicknesses between 160 and 300 nm were fabricated by focused ion beam (FIB) milling and lifted out from bulk single crystals, following the procedure described in Ref. [17]. The [100]_pc_ oriented samples were investigated by in situ STEM during controlled heating and cooling from RT through *T_C_* (∼550°C). In this study, the sample thickness lies along the electron beam direction, with DWs extending through this thickness. Using a DENSsolutions Climate in situ holder, all samples were heat‐cycled from RT to 600°C and back at 0.33°C s^−^^1^ (see Methods). STEM dark field (STEM‐DF) images were acquired at fixed temperature intervals during cooling.

**FIGURE 1:**
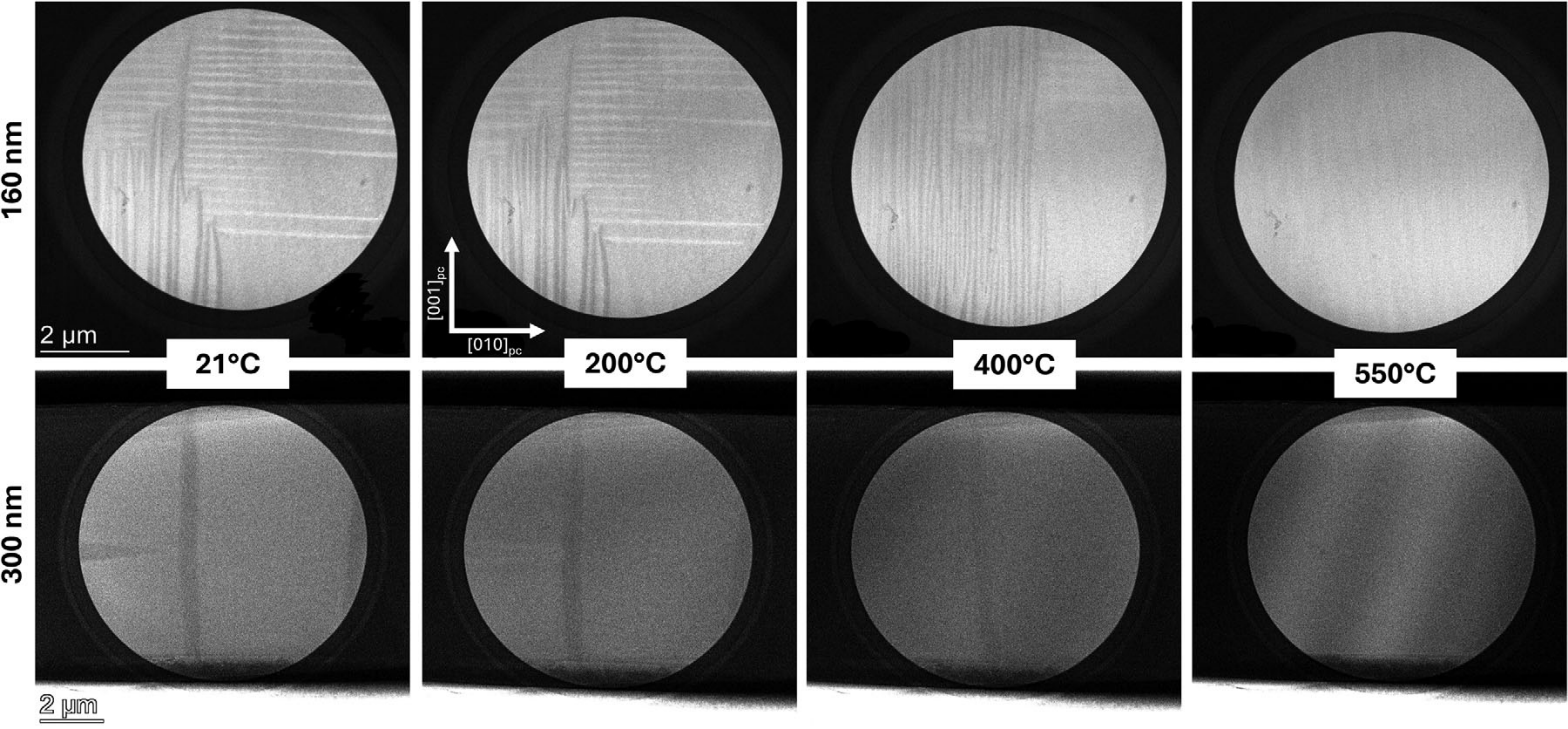
Evolution of the domain wall (DW) structure during cooling from *T_C_*. Representative scanning transmission electron microscopy dark field (STEM‐DF) images showing domain structure in 160 and 300 nm thick LaAlO_3_ samples at selected temperatures between 550°C and room temperature. The 160 nm sample exhibits a dense domain configuration with highly curved DWs, while the 300 nm sample displays comparatively fewer and straighter DWs.

Figure 1 displays representative STEM‐DF images at selected temperatures, with the full dataset provided in (Figures S1, S2, and S3). The observed domains correspond to the commonly reported LAO domain variants, [001]_pc_ and [010]_pc_ [19, 20]. For this work, 10 samples were imaged at intervals of 50°C between *T_C_* and RT. Manual analysis of large in situ datasets, particularly STEM images, is inherently challenging due to the high volume of images and is a very time‐consuming task that can be plagued with human error. Machine learning‐based image segmentation offers a promising alternative method, enabling more accurate analysis in a vastly reduced timeframe. Fully convolutional neural networks (FCNNs) such as U‐Net [21] have become popular within fields such as biomedical science [22] and have recently shown increasing use in materials science [23, 24]. These models are capable of semantic segmentation (the classification of each individual pixel in an image) while requiring only a small set of hand‐labeled training data.

**FIGURE 2:**
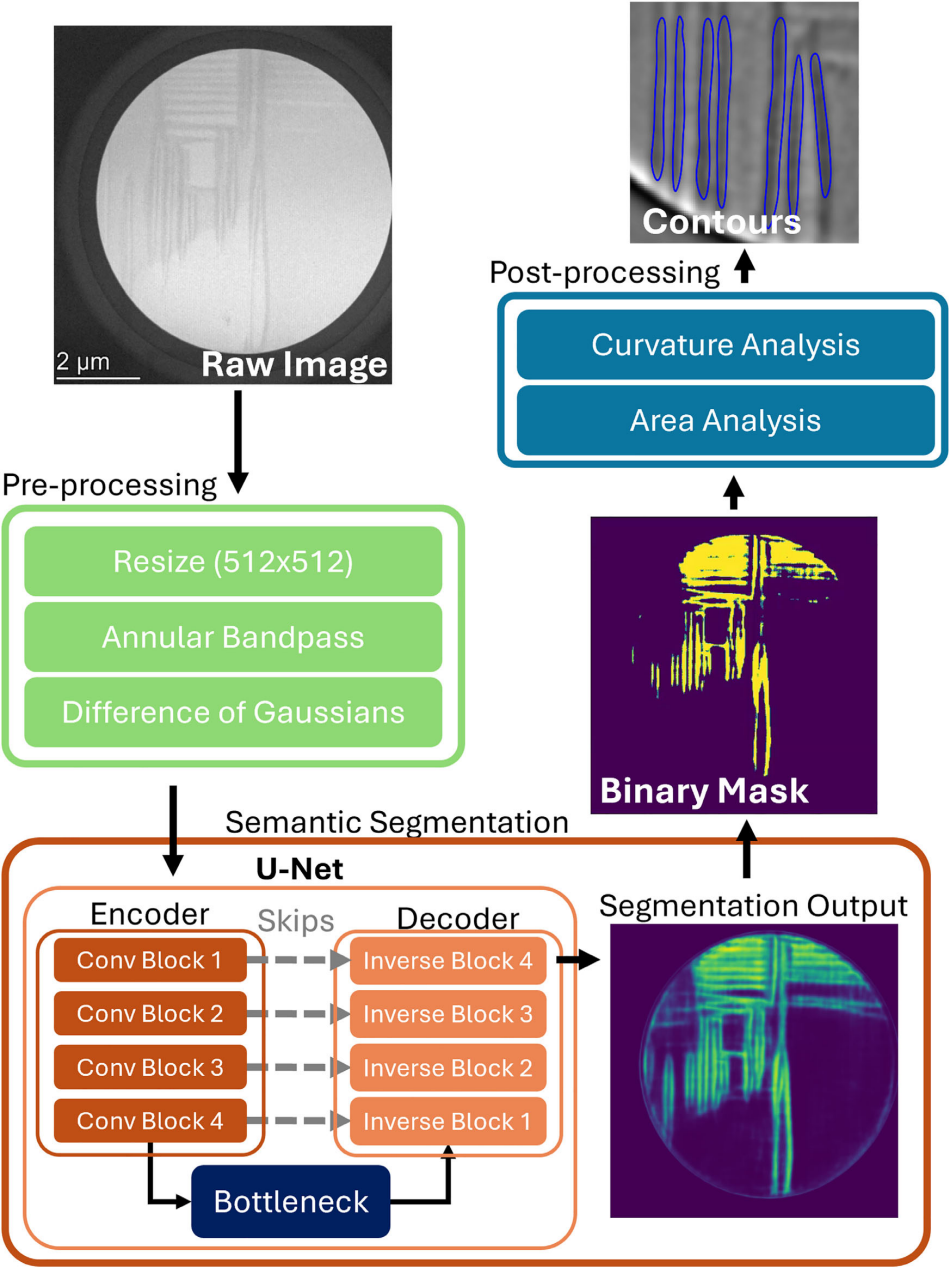
Machine learning workflow used to calculate domain wall (DW) curvature and area fraction from scanning transmission electron microscopy dark field (STEM‐DF) images. Raw STEM‐DF images undergo filtered preprocessing to eliminate image artifacts before semantic segmentation is carried out using U‐NET. The resulting segmentation output is converted into a binary mask, from which DW contours are extracted. These contours are subsequently used to calculate the DW area fraction and curvature.

Here, a machine‐learning model was trained to identify DWs, enabling a more robust quantification of DW area fraction and curvature as a function of temperature. The model is based on U‐Net [21] with a ResNet‐34 encoder [25]; the workflow is illustrated in Figure 2. A representative subset of images (three images per sample, ≈25% of the dataset) was randomly selected and manually annotated pixel‐wise into three classes: DWs, domains, and background (regions outside the transparent window of the MEMS in situ chip) using the open‐source data annotation software Label Studio [26]. Of these annotated images, 75% were used for training, and the remaining 25% were reserved as a ground‐truth validation set.

The model performs semantic segmentation to classify each pixel as DW, domain, or background (Figure S4 shows representative DW predictions output by the trained model). Predicted DW masks were used to compute DW area (a representation of DW density), and contiguous DW pixels in representative cropped regions of the image were fitted with splines to extract local curvature. DW area and curvature were averaged across all samples with a given thickness at each temperature interval to yield mean values for each sample thickness set. Additional details regarding DW area fraction and curvature calculation are provided in the SI.

**FIGURE 3:**
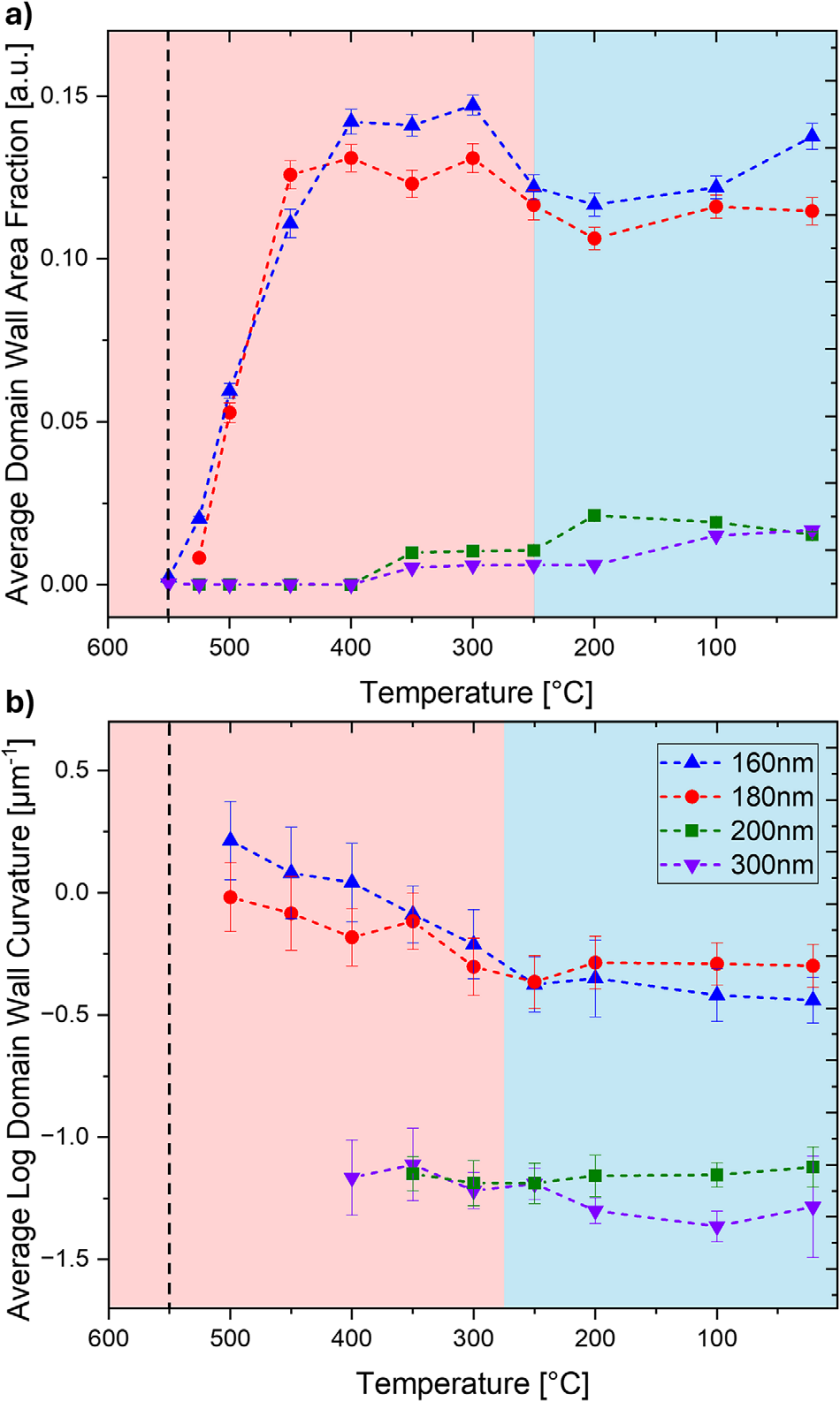
Temperature‐dependent evolution of average a) domain wall (DW) area fraction and b) log of DW curvature during cooling from *T_C_* (represented by the black dashed line) across thicknesses. The red and blue shaded regions indicate the temperature ranges associated with the super‐elastic and freezing regimes. Uncertainties derived using bootstrapping, with uncertainties for 200 and 300 nm domain wall area fraction ∼1 × 10^−4^.

The machine learning analysis revealed pronounced thickness‐dependent differences in DW morphology and behavior during cooling. Figure 3 compares the mean DW area fraction and mean log(curvature) during cooling for all sample thicknesses studied. For thin samples (160–180 nm), the DW area fraction significantly increases just below *T_C_*, reaching a maximum between 300°C and 400°C before gradually decreasing and plateauing at ≈200°C.

It is important to note that, as seen in the DF STEM images in the SI (Figures S1, S2, and S3), DW contrast becomes weaker at high temperatures, particularly ∼500°C and above, which makes DWs more difficult to identify and quantify reliably. Therefore, the apparent reduction in measured DW area near *T_C_* may partly reflect reduced image contrast and detectability rather than a physical reduction in DW density.

Samples in the 200–300 nm crossover regime exhibited a nearly constant, low DW area fraction across all temperatures, with small, discrete increases that correspond to the formation of individual DWs, indicating strongly suppressed nucleation and mobility.

A similar trend was observed for DW curvature. In the thinner samples, DWs exhibit a high curvature value of ∼1–1.5 µm^−1^ near *T_C_*, which decreases continuously on cooling and plateaus at ≈250°C with curvatures in the range ∼0.3–0.5 µm^−1^ (Figure S6 shows a comparison of DW area fraction and curvature for 160–180 nm samples). Thicker samples displayed lower curvature by approximately an order of magnitude throughout the entire cooling cycle, suggesting suppressed nucleation, pinned walls, and mobility.

This thickness‐dependent evolution follows the previously established monopolar–dipolar crossover framework, highlighting the importance of elastic interactions in governing DW behavior over a wide temperature range. The increased curvature observed in thinner samples is attributed to enhanced elastic relaxation of kink‐induced strain fields, which becomes more pronounced as the system size decreases, in agreement with the interaction regimes described by Lu et al. [16] and experimentally confirmed by Scott et al. [17], demonstrating the existence of dipolar, monopolar, and crossover regimes at RT for LAO. In thin samples, the high density of DWs leads to strong domain–domain interactions, which can influence their morphology and dynamics, as reported in other ferroic systems such as BaTiO_3_ [27]. The model of Lu et al. [16] describes a pure ferroelastic system where DWs extend through the thickness (*t*) and the wall‐wall separation (*d*) is defined in‐plane. The model predicts a transition from dipolar (∝ *d^−^^2^*) to monopolar (∝ *d^−^^1^*) interactions with increasing system size, where such interactions originate from kink‐mediated elastic strain fields [15, 16, 28]. In the dipolar regime, strong strain deformations are observed near the surface, giving rise to highly curved and mobile DWs. As the system size increases, these strain deformations decay and almost disappear (monopolar regime), amplifying the pinning of DWs, penalizing curvature and favoring straight DWs.

The present work uses the same samples as in the work of Scott et al. [17] under identical imaging conditions and extends it to include thermal behavior. Samples with thicknesses of 160−180 nm fall within the dipolar regime and demonstrate significantly different temperature‐dependent DW behavior compared to 200–300 nm thick samples (crossover regime). This demonstrates that relatively small changes in thickness can produce relatively large changes in DW behavior and aligns with the changes in DW configuration found in samples of the same thicknesses at RT in previous work [17]. Our experimental findings are consistent with theoretical observations and provide, to the best of our knowledge, the first experimental evidence of the temperature‐driven evolution of DWs across the size‐dependent crossover regime. That is, the monopolar, dipolar, and crossover regimes are determined by the system size (thickness), while temperature modulates DW behavior within a given regime.

As a final point regarding curvature, the observed increase in DW curvature at elevated temperatures should not be interpreted as evidence of an increase in spontaneous strain, which is expected to decrease as the sample approaches T_C_. Previous work has shown that ferroelastic DWs can exhibit complex morphologies involving kinks, local bending, and wall meandering, arising from long‐range elastic interactions and local relaxation processes [16, 29, 30]. In this context, we interpret the increased curvature observed in the thinner samples as being consistent with DW reconfiguration within the dipolar interaction regime previously identified in LAO. However, the origin of the temperature dependence of DW curvature remains unclear, and we propose that a theoretical model linking temperature, spontaneous strain, and DW curvature would be an important direction for future investigation.

The temperature‐dependent DW behavior observed here resembles the superelastic and freezing regimes reported for bulk LAO [18, 31], where dynamic mechanical analysis revealed strong anelastic softening between *T_C_* (∼545°C) and 300°C, corresponding to the superelastic regime, followed by a marked reduction in DW activity below ∼250°C (freezing regime), where defect pinning dominates. As seen in Figure 3, the DW curvature and area fraction of 160–180 nm samples (dipolar regime) vary significantly between 550°C and 300°C but remain mostly static at temperatures below ∼250°C. The significant changes in DW configuration at higher temperatures are also exhibited in Figures S1 and S2. These observations suggest that samples within the dipolar regime may exhibit higher DW reconfiguration at elevated temperatures and increasingly reduced domain reconfiguration at lower temperatures. This behavior qualitatively resembles the reported superelastic and freezing regimes reported for bulk LAO. In contrast, samples within the crossover regime remain largely unchanged throughout cooling.

**FIGURE 4:**
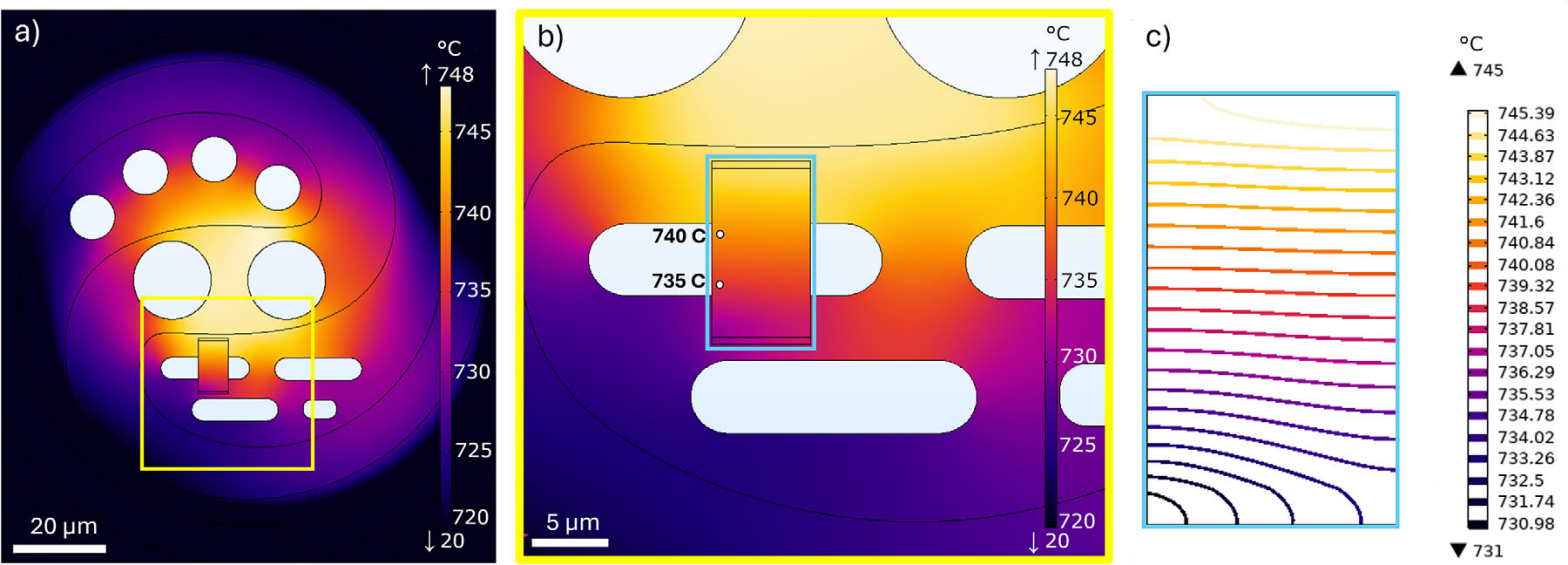
COMSOL simulation of thermal gradients in the in situ MEMS heating geometry. (a) Temperature distribution across the microheater window at 747°C (1.2 V input), indicating a difference of 10 ± 3°C between the inner and outer windows. (b) Temperature variation across an LaAlO_3_ sample positioned on a window, showing a thermal gradient of 5 ± 3°C from one edge of the window to the other. (c) Thermal gradient across the sample's width (including areas away from the window); the total gradient is ∼12°C.

Interestingly, in the crossover regime (200–300 nm), domains appeared only at temperatures of ≈400°C and below, suggesting a subtle thickness‐induced shift in *T_C_*. Such shifts may arise from strain confinement or finite‐size effects that alter the balance of elastic contributions to the free energy. To evaluate this apparent shift in transition temperature and the possible effect of thermal gradients or mechanical bulging from the in situ heating geometry, COMSOL finite‐element simulations were performed (Figure 4). Figure S7 and Table S1 show model geometry and sample parameters used.

These simulations indicate a temperature gradient of ∼5 ± 3°C across the MEMS window and ∼12 ± 3°C across the full sample (Figure 4), together with ∼5 µm out‐of‐plane bulging at set temperatures as high as 747°C (Figure S8). To represent a worst‐case scenario, simulations were performed at 747°C, exceeding the maximum experimental temperature (600°C). Under these conditions, both temperature gradients and membrane deformation remain relatively small and spatially uniform, indicating that thermal nonuniformity is expected to be equal or lower under experimental conditions. Figure S8 further evaluates membrane deformation using the experimental MEMS chip geometry, demonstrating that even at elevated temperatures (up to 747°C), out‐of‐plane bulging shows minimal variation with sample thickness. Importantly, the magnitude of the simulated temperature gradients is insufficient to account for the observed shift in *T_C_*, which only occurs in thicker samples (200–300 nm), within the crossover regime. This indicates that the observed shift in *T_C_* cannot be attributed to thermal inhomogeneity or mechanical deformation but instead reflects an intrinsic, thickness‐dependent effect.

To assess the potential role of oxygen vacancies in the observed behavior, energy dispersive X‐ray spectroscopy (EDX) scans were acquired before and after heating each sample. A representative example for a 160 nm sample is shown in Figure S9. No significant change in the oxygen atomic fraction is observed between heating cycles. Additionally, DW behavior (curvature and density) is reproducible within thickness sets and upon repeated heating and cooling. While the formation of point defects under vacuum cannot be entirely excluded, the absence of significant compositional changes together with the observed reversibility of the domain structure suggests that defect formation, such as oxygen vacancies, is not the dominant factor governing the observed thickness‐ and temperature‐dependent behaviors.

The DW curvature and density variations observed here have direct implications for nanoscale functionality. Vasudevan et al. demonstrated that curvature modulates conductivity in BiFeO_3_, with local DW bending enhancing carrier accumulation [29]. Similarly, studies in ErMnO_3_ revealed that DW bending introduces highly charged DW segments that modulate local electric fields, resulting in conductivity variations at the nanoscale [30]. These studies establish a clear link between DW geometry and charge transport. By analogy, curvature‐dependent strain fields in ferroelastics such as LAO could influence ionic or phonon transport, suggesting a route to further tune local strain for thermal or electromechanical properties. This highlights curvature, controlled through both thickness and temperature, as a powerful parameter for designing emergent functionality in ferroelastic membranes.

## 3 Conclusion

This work investigates LaAlO_3_ as a model system for understanding the interplay between thermal activation, elastic relaxation, and size‐dependent DW interactions in ferroelastic materials. It is demonstrated that temperature and thickness act as coupled parameters that can govern DW morphology and evolution in free‐standing ferroelastics.

Overall, thicker samples (200–300 nm) exhibited simpler domain configurations, with reduced DW area fraction and fewer junctions, with straighter DWs. Thinner samples (<200 nm) displayed more complex configurations, with a higher DW area fraction, a higher number of junctions, and DW curvature that increased at temperatures near *T_C_*. Thinner, dipolar samples continue to exhibit significant DW evolution throughout cooling, while in thicker, crossover regime samples, DWs remain comparatively static.

While the behavior observed here is specific to LAO, the underlying concepts of elastic boundary conditions and thickness‐controlled DW dynamics are expected to be relevant across a broad range of ferroelastic systems where domain evolution is governed by a similar interplay between elastic interactions, defect pinning, and thermal effects. The present results therefore provide a framework for future investigations of temperature‐dependent DW dynamics in other ferroelastic and multiferroic materials where elastic interactions compete with additional order parameters. Future studies combining time‐resolved in situ diffraction, acoustic spectroscopy, and phase‐field simulations could further elucidate how elastic softening and defect pinning collectively define DW dynamics and functional properties at the nanoscale.

## 4 Experimental Section/Methods

Free‐standing samples of ≈7 µm × 12 µm, with thicknesses ranging from 160 to 300 nm, were prepared using a TESCAN Lyra 3 dual beam FIB/SEM by standard focused ion beam (FIB) milling techniques. These samples were fabricated to have posts on either side, similar to previous in situ heating TEM studies [32], to prevent direct contact with the electron‐transparent window during heating and resemble free‐standing conditions, and were placed on an in situ heating MEMS chips via conventional ex situ lift out, fully detailed in Ref. [16]. 10 samples were analyzed in this study: 3 samples of 160 nm, 5 samples of 180 nm, 1 sample of 200 nm, and 1 sample of 300 nm.

STEM images were recorded using a dark field STEM (STEM‐DF) detector on an FEI TALOS F200 G2 at 200 kV, with a dwell time of 20 µs.

All samples were heat cycled from RT to 600°C (above *T_C_* ∼550°C) and back at a rate of 0.33°C s^−1^, using a DENSsolutions Climate system with an open cell configuration, as described elsewhere [17].

Image analysis was carried out using a U‐NET [21] model with a ResNet‐34 [25] encoder. The model was trained using manually labeled images, 33 of which were used as a training set and another 9 images used as the ground‐truth validation set. All manual labeling was performed using the open‐source data annotation software Label Studio [26]. Prior to training, images were pre‐processed with spatial filters (difference‐of‐Gaussians and annular bandpass) to suppress imaging artefacts as much as possible, most notably bending contours. Bending contours are large regions of alternating dark and light contrast that arise from thermal expansion of the samples and the in situ MEMS chip; the applied filters minimized these features to reduce their influence on model performance. The loss function was the mean of binary cross‐entropy (BCE) and Dice loss. Model hyperparameters were learning rate *η* = 1×10^−^^4^, batch size *|B|* = 2, and class weighting for DW:domain:background = 60:30:10. The model achieved a final test loss of 0.057 (see training curve in Figure S5).

## Funding

EPSRC PIADS studentship (grant no: EP/S023321/1), ORNL CNMS user project (proposal number: CNMS2024‐B‐02660)

## Conflicts of Interest

The authors disclose no conflicts of interest.

## Supporting information




**Supporting File**: smll74540‐sup‐0001‐SuppMat.docx.

## Data Availability

The code used for this study can be accessed here: https://github.com/lged01/LaAlO3‐Segmentation‐Network. The training data used, as well as the final model, is available here: https://www.dropbox.com/scl/fo/a86c23bf74xkc9l6lqdd6/AMJ66EC8MvMy0o8Rc6VDDu4?rlkey=26tx9n36njy09sgjo2osb6o0q&st=9i9eymcl&dl=0.

## References

[1] E. K. H. Salje and G. Lu , “Introduction to Domain Boundary Engineering,” in Domain Walls: From Fundamental Properties to Nanotechnology Concepts, ed. D Meier , J Seidel , M Gregg , and R Ramesh (Oxford University Press, 2020), 10.1093/oso/9780198862499.003.0005.

[2] J. F. Scott , E. K. H. Salje , and M. A. Carpenter , “Domain Wall Damping and Elastic Softening in SrTiO_3_: Evidence for Polar Twin Walls,” Physical Review Letters 109, no. 18 (2012): 187601, 10.1103/PhysRevLett.109.187601. 23215329

[3] E. K. H. Salje , O. Aktas , M. A. Carpenter , V. V. Laguta , and J. F. Scott , “Domains Within Domains and Walls Within Walls: Evidence for Polar Domains in Cryogenic SrTiO_3_ ,” Physical Review Letters 111, no. 24 (2013): 247603, 10.1103/PhysRevLett.111.247603. 24483700

[4] D. Meier , J. Seidel , A. Cano , et al., “Anisotropic Conductance at Improper Ferroelectric Domain Walls,” Nature Materials 11, no. 4 (2012): 284–288, 10.1038/nmat3249. 22367003

[5] J. Seidel , L. W. Martin , Q. He , et al., “Conduction at Domain Walls in Oxide Multiferroics,” Nature Materials 8, no. 3 (2009): 229–234, 10.1038/nmat2373. 19169247

[6] E. K. H. Salje and H. Zhang , “Domain Boundary Engineering,” Phase Transitions 82, no. 6 (2009): 452–469, 10.1080/01411590902936138.

[7] E. K. H. Salje , “Multiferroic Domain Boundaries as Active Memory Devices: Trajectories Towards Domain Boundary Engineering,” Chemphyschem 11, no. 5 (2010): 940–950, 10.1002/cphc.200900943. 20217888

[8] G. F. Nataf , M. Guennou , J. M. Gregg , et al., “Domain‐Wall Engineering and Topological Defects in Ferroelectric and Ferroelastic Materials,” Nature Reviews Physics 2, no. 11 (2020): 634–648, 10.1038/s42254-020-0235-z.

[9] D. Wang , S. Hao , B. Dkhil , B. Tian , and C. Duan , “Ferroelectric Materials for Neuroinspired Computing Applications,” Fundamental Research 4, no. 5 (2024): 1272–1291, 10.1016/j.fmre.2023.04.013. 39431127 PMC11489484

[10] P. Sharma and J. Seidel , “Neuromorphic Functionality of Ferroelectric Domain Walls,” Neuromorphic Computing and Engineering 3, no. 2 (2023): 022001, 10.1088/2634-4386/accfbb.

[11] E. K. H. Salje , “Ferroelastic Domain Walls as Templates for Multiferroic Devices,” Journal of Applied Physics 128, no. 16 (2020): 164104, 10.1063/5.0029160.

[12] M. M. Vopson , “Fundamentals of Multiferroic Materials and Their Possible Applications,” Critical Reviews in Solid State and Materials Sciences 40, no. 4 (2015): 223–250, 10.1080/10408436.2014.992584.

[13] S. D. Ha and S. Ramanathan , “Adaptive Oxide Electronics: A Review,” Journal of Applied Physics 110, no. 7 (2011): 071101, 10.1063/1.3640806.

[14] E. K. H. Salje , “Ferroelastic Twinning in Minerals: A Source of Trace Elements, Conductivity, and Unexpected Piezoelectricity,” Minerals 11, no. 5 (2021): 478, 10.3390/min11050478.

[15] G. Lu , X. Ding , J. Sun , and E. K. H. Salje , “Wall‐Wall and Kink‐Kink Interactions in Ferroelastic Materials,” Physical Review B 106, no. 14 (2022): 144105, 10.1103/PhysRevB.106.144105.

[16] G. Lu , K. Hideo , X. Ding , et al., “Influence of Kinks on the Interaction Energy between Ferroelastic Domain Walls in Membranes and Thin Films,” microstructures 3 (2023): 2023033, 10.20517/microstructures.2023.28.

[17] J. J. R. Scott , G. Lu , B. J. Rodriguez , I. MacLaren , E. K. H. Salje , and M. Arredondo , “Evidence of the Monopolar‐Dipolar Crossover Regime: A Multiscale Study of Ferroelastic Domains by in Situ Microscopy Techniques,” Small 20, no. 35 (2024): 2400646, 10.1002/smll.202400646. 38686673

[18] R. J. Harrison and S. A. T. Redfern , “The Influence of Transformation Twins on the Seismic‐Frequency Elastic and Anelastic Properties of Perovskite: Dynamical Mechanical Analysis of Single Crystal LaAlO3,” Physics of the Earth and Planetary Interiors 134, no. 3 (2002): 253–272, 10.1016/S0031-9201(02)00190-5.

[19] J. J. R. Scott , B. Casals , K. F. Luo , et al., “Avalanche Criticality in LaAlO$$_3$$ and the Effect of Aspect Ratio,” Scientific Reports 12, no. 1 (2022): 14818, 10.1038/s41598-022-18390-7. 36050337 PMC9437108

[20] C. H. Kim , J. W. Jang , S. Y. Cho , I. T. Kim , and K. S. Hong , “Ferroelastic Twins in LaAlO3 Polycrystals,” Physica B: Condensed Matter 262, no. 3 (1999): 438–443, 10.1016/S0921-4526(98)00848-5.

[21] O. Ronneberger , P. Fischer , and T. Brox , “U‐Net: Convolutional Networks for Biomedical Image Segmentation,” in Medical Image Computing and Computer‐Assisted Intervention—MICCAI 2015, ed. N. Navab , J. Hornegger , W. M. Wells , and A. F. Frangi (Springer International Publishing, 2015), 234–241, 10.1007/978-3-319-24574-4_28.

[22] R. Azad , E. K. Aghdam , A. Rauland , et al., “Medical Image Segmentation Review: The Success of U‐Net,” IEEE Transactions on Pattern Analysis and Machine Intelligence 46, no. 12 (2024): 10076–10095, 10.1109/TPAMI.2024.3435571. 39167505

[23] X. Lucy Lu and K. He , “Machine Learning Enhanced Image Segmentation for High‐Fidelity STEM Data Analysis,” Microanalysis 29, no. S1 (2023): 1895–1896, 10.1093/micmic/ozad067.978.

[24] A. C. Houston , S. B. Harris , H. Wang , et al., “Atom Identification in Bilayer Moiré Materials with Gomb‐Net,” Nano Letters 25, no. 23 (2025): 9277–9284, 10.1021/acs.nanolett.5c01460. 40454431

[25] K. He , X. Zhang , S. Ren , and J. Sun , “Deep Residual Learning for Image Recognition,” in Proceedings of the IEEE Conference on Computer Vision and Pattern Recognition (2025), 770–778, Accessed October 6. https://openaccess.thecvf.com/content_cvpr_2016/html/He_Deep_Residual_Learning_CVPR_2016_paper.html.

[26] M. Tkachenko , M. Malyuk , A. Holmanyuk , and N. Liubimov , “Label Studio: Data Labelling Software,” (GitHub, 2025), https://github.com/HumanSignal/label‐studio.

[27] R. Ignatans , D. Damjanovic , and V. Tileli , “Individual Barkhausen Pulses of Ferroelastic Nanodomains,” Physical Review Letters 127, no. 16 (2021): 167601, 10.1103/PhysRevLett.127.167601. 34723579

[28] X. He , S. Li , X. Ding , J. Sun , S. M. Selbach , and E. K. H. Salje , “The Interaction Between Vacancies and Twin Walls, Junctions, and Kinks, and Their Mechanical Properties in Ferroelastic Materials,” Acta Materialia 178 (2019): 26–35, 10.1016/j.actamat.2019.07.051.

[29] R. K. Vasudevan , A. N. Morozovska , E. A. Eliseev , et al., “Domain Wall Geometry Controls Conduction in Ferroelectrics,” Nano Letters 12, no. 11 (2012): 5524–5531, 10.1021/nl302382k. 22994244

[30] E. D. Roede , K. Shapovalov , T. J. Moran , et al., “The Third Dimension of Ferroelectric Domain Walls,” Advanced Materials 34, no. 36 (2022): 2202614, 10.1002/adma.202202614. 35820118

[31] R. J. Harrison , S. A. T. Redfern , and E. K. H. Salje , “Dynamical Excitation and Anelastic Relaxation of Ferroelastic Domain Walls in LaAlO3,” Physical Review B 69, no. 14 (2004): 144101, 10.1103/PhysRevB.69.144101.

[32] T. O'Reilly , K. M. Holsgrove , X. Zhang , et al., “The Effect of Chemical Environment and Temperature on the Domain Structure of Free‐Standing BaTiO_3_ via in Situ STEM,” Advanced Science 10, no. 29 (2023): 2303028, 10.1002/advs.202303028. 37607120 PMC10582436

