# Supplementary material for: In Situ Heating STEM of the Thickness‐Dependent Evolution of Ferroelastic Domains
Source: Small. 2026 Jul 14;22(50):e74540. doi: 10.1002/smll.74540 (PMC13548898; doi:10.1002/smll.74540)
Supplement: Supplementary file 1 [file SMLL-22-e74540-s001.docx]

**Supporting Information for “In situ heating STEM of the Thickness-Dependent Evolution of Ferroelastic Domains”**

*Luka Geddis Zellmann*, Sumner B. Harris, John R. R. Scott, Yi-Chieh Yang, Joerg R. Jinschek, Rama K. Vasudevan, Miryam Arredondo**

L. Geddis Zellmann, J. R. R. Scott, M. Arredondo

School of Mathematics and Physics, Queen’s University Belfast, Belfast, UK

Email: [lgeddiszellmann01@qub.ac.uk](mailto:lgeddiszellmann01@qub.ac.uk), [m.arredondo@qub.ac.uk](mailto:m.arredondo@qub.ac.uk)

S. B. Harris, R. K. Vasudevan

Center for Nanophase Materials Sciences (CNMS), Oak Ridge National Laboratory (ORNL), Oak Ridge, USA

Y.-C. Yang, J. R. Jinschek

National Center for Nano Fabrication and Characterization (DTU Nanolab), Technical University of Denmark, Kgs. Lyngby, Denmark

I. **Machine Learning Analysis**

Outputs of the U-NET model must be manually tweaked for an accurate and reliable calculation of domain wall (DW) curvature and area. Adjustments can be made to the model output when converting from the raw output to the binary maps which are used for quantification by altering confidence threshold - decreasing the confidence threshold allows for more domain walls to be captured, but introduces more noise to the binary map which can result in domains merging together, while increasing the confidence threshold can eliminate noise but results in the loss of smaller domain walls. Therefore, two different approaches were used in order to obtain accurate values for the DW area and curvature.

a. **Domain Wall Area**

For the calculation of DW area, a bootstrapping technique was employed. An upper and lower limit to the confidence threshold was manually determined for each image – the upper threshold was determined as the point where no noise could be seen, and the lower threshold was the point where all DWs were represented. This was performed interactively using a basic graphical user interface to maximize efficiency. 100 binary maps were generated at evenly spaced confidence threshold intervals between these limits to serve as bootstrap samples, the DW area was calculated for each one, and bootstrapping was used to calculate a mean DW area and an uncertainty for each image. This allowed for an accurate calculation of the DW area while overcoming the limitations of the U-NET model and quantifying the uncertainty inherent in the process.

b. **Domain Wall Curvature**

For curvature, a different approach was required as even slight changes in the binary map can lead to significant effects on the calculated curvature. Therefore, the U-NET outputs were manually cropped to smaller, representative areas of the image in which U-NET was able to identify all domain walls accurately, rather than calculating curvature on every domain wall in a given image. This allowed regions with reduced accuracy of predictions to be neglected to prevent any impact on the results. The confidence threshold was then set manually prior to curvature calculation.

In order to calculate the curvature of domain walls, the measure.find_contours() function from the scikit-image python library was applied to the binary masks created from the U-NET output to fit contours to all DWs. These contours are then interpolated as plane curves, before using equation 1 to calculate the curvature at every point along the path of the curve.

Equation 1: $\kappa= \frac{\left| x^{'}y^{''}- x^{''}y^{'} \right|}{{(\left( x^{'} \right)^{2}+ {(y^{'})}^{2})}^{\frac{3}{2}}}$

In equation 1, $\kappa$ represents the curvature, $x^{'}$ represents the first derivative of $x$, $x^{''}$ represents the second derivative of $x$, $y^{'}$ represents the first derivative of $y$, and $y^{''}$ represents the second derivative of $y$.

The curvature at the ends of the contour (where the contour does not match the domain wall) is neglected as the median curvature for the contour is found. Bootstrapping is then used across all contours within the image to find an average curvature value and uncertainty.

II. **Supplementary Figures and Tables**

**Figure S1**. Scanning transmission electron microscopy dark field (STEM-DF) images of three 160 nm free-standing LaAlO₃ samples during cooling from 550 °C to room temperature, showing dense, curved, and highly mobile domain walls near T_C_ that gradually straighten and freeze as temperature decreases.

**Figure S2**. Scanning transmission electron microscopy dark field (STEM-DF)images of five 180 nm free-standing LaAlO₃ samples during cooling from 550 °C to room temperature, revealing the progressive straightening and reduced density of domain walls as the system transitions from the super-elastic to freezing regime in the crossover thickness range.

**Figure S3**. Scanning transmission electron microscopy dark field (STEM-DF) images of 200 nm and 300 nm free-standing LaAlO₃ samples during cooling from 550 °C to room temperature, showing static, low-curvature domain walls.

**Figure S4.** Scanning transmission electron microscopy (STEM) images of an a) 160 nm and b) 180 nm LaAlO_3_ sample at 400 °C and 21 °C, alongside segmentation outputs from the trained U-NET model showing the predicted positions of domain walls (DWs). Even when contrast is significantly affected by bending contours, as in the 400 °C images, the model is able to identify DWs accurately.

**Figure S5.** Training curve showing the evolution of the combined binary cross-entropy (BCE) and Dice loss during model optimization for domain wall segmentation. The steady decrease and plateau of the loss function indicate stable convergence and robust performance of the U-Net (ResNet-34 encoder) model.

**Figure S6.** Temperature-dependent evolution of normalized domain wall area and curvature in (a) 160 nm and (b) 180 nm free-standing LaAlO₃ (LAO). Both samples exhibit increased domain wall area fraction and high curvature near T_C_, followed by progressive straightening and slightly reduced area fraction as the system cools towards room temperature. The red and blue shaded regions indicate the temperature ranges associated with the super-elastic and freezing regimes.

**Figure S7.** COMSOL model based on scanning electron microscopy (SEM) measurements of a free-standing LaAlO₃ (LAO) sample on a DENSsolutions in situ heating chip. (a) SEM image of the experimental configuration, (b) corresponding COMSOL geometry used for simulations (based on a DENSsolutions Wildfire chip), and (c) modelled LAO sample with identical dimensions to the experimental samples.

| **Property** | **Value** | **Unit** |
| --- | --- | --- |
| Coefficient of Thermal Expansion | 10x10^-6^ | 1/K |
| Heat Capacity at Constant Pressure | 730 | J(kg*K) |
| Density | 6.56 | g/cm^3^ |
| Thermal Conductivity | 10 | W(m*K) |
| Young’s Modulus | 400x10^9^ | Pa |
| Poisson’s Ratio | 0.22 | No Units |
| Electrical Conductivity | 0 | S/m |
| Relative Permittivity | 5.7 | No Units |

**Table S1.** LaAlO_3_ parameters used in the COMSOL simulations.

**Figure S8.** COMSOL simulation of the bulging experienced by the a) microheater membrane and b) LAO sample (outlined in white) at 747ºC (input = 1.2V). The overall bulging experienced by the sample is approximately 5μm. c) Out-of-plane displacement due to bulging across the lengths of the sample for samples of varying thickness at several temperatures. Bulging is greater at higher temperatures and thinner samples experience slightly greater bulging than thicker samples at the same temperature.

**Figure S9.** Energy dispersive X-ray spectroscopy (EDX) atomic percentage profiles for oxygen, acquired before and after annealing under ultra-high vacuum for a 160 nm LaAlO_3_ sample. No significant change in the atomic percentage of oxygen is detected.
